# SHARPIN stabilizes estrogen receptor α and promotes breast cancer cell proliferation

**DOI:** 10.18632/oncotarget.20368

**Published:** 2017-08-19

**Authors:** Ting Zhuang, Sifan Yu, Lichen Zhang, Huijie Yang, Xin Li, Yingxiang Hou, Zhenhua Liu, Yuanyuan Shi, Weilong Wang, Na Yu, Anqi Li, Xuefeng Li, Xiumin Li, Gang Niu, Juntao Xu, Muhammad Sharif Hasni, Kun Mu, Hui Wang, Jian Zhu

**Affiliations:** ^1^ Research Center for Immunology, School of Laboratory Medicine, Xinxiang Medical University, Xinxiang, Henan, China; ^2^ Henan Collaborative Innovation Center of Molecular Diagnosis and Laboratory Medicine, Xinxiang Medical University, Xinxiang, Henan, China; ^3^ Key Laboratory of Carcinogenesis and Translational Research, Ministry of Education, Department of Renal Cancer and Melanoma, Peking University School of Oncology, Beijing Cancer Hospital and Institute, Beijing, China; ^4^ Laboratory of Genetic Regulators in the Immune System, School of Laboratory Medicine, Xinxiang Medical University, Xinxiang, Henan, China; ^5^ Synthetic Biology Remaking Engineering and Application Laboratory, School of Life Sciences and Technology, Xinxiang Medical University, Xinxiang, Henan, China; ^6^ Department of Gastroenterology, The Third Affiliated Hospital of Xinxiang Medical University, Xinxiang, Henan, China; ^7^ Center for Cancer Research, Xinxiang Medical University, Xinxiang, Henan, China; ^8^ School of International Education, Xinxiang Medical University, Xinxiang, Henan, China; ^9^ Department of Medical Oncology, The First Affiliated Hospital, University of South China, Hengyang, Hunan, China; ^10^ Department of Cancer genomics, LemonData biotech (Shenzhen) Ltd, Shenzhen, Guangdong, China; ^11^ Phil Rivers Technology (Beijing) Ltd. Beijing, China; ^12^ Institute of Biochemistry University of Balochistan, Quetta, Pakistan; ^13^ Department of Hematology and Transfusion Medicine, Lund University, Lund, Sweden; ^14^ Department of Pathology, School of Medicine, Shandong University, Jinan, Shandong, China; ^15^ Department of Molecular Biology, University of Texas Southwestern Medical Center, Dallas, Texas, USA

**Keywords:** SHARPIN, ER alpha, breast cancer, ubiquitination, protein stability

## Abstract

Estrogen receptor α is expressed in the majority of breast cancers and promotes estrogen-dependent cancer progression. In our study, we identified the novel E3 ubiquitin ligase SHARPIN function to facilitate ERα signaling. SHARPIN is highly expressed in human breast cancer and correlates with ERα protein level by immunohistochemistry. SHARPIN expression level correlates with poor prognosis in ERα positive breast cancer patients. SHARPIN depletion based RNA-sequence data shows that ERα signaling is a potential SHARPIN target. SHARPIN depletion significantly decreases ERα protein level, ERα target genes expression and estrogen response element activity in breast cancer cells, while SHARPIN overexpression could reverse these effects. SHARPIN depletion significantly decreases estrogen stimulated cell proliferation in breast cancer cells, which effect could be further rescued by ERα overexpression. Further mechanistic study reveals that SHARPIN mainly localizes in the cytosol and interacts with ERα both in the cytosol and the nuclear. SHARPIN regulates ERα signaling through protein stability, not through gene expression. SHARPIN stabilizes ERα protein via prohibiting ERα protein poly-ubiquitination. Further study shows that SHARPIN could facilitate the mono-ubiquitinaiton of ERα at K302/303 sites and facilitate ERE luciferase activity. Together, our findings propose a novel ERα modulation mechanism in supporting breast cancer cell growth, in which SHARPIN could be one suitable target for development of novel therapy for ERα positive breast cancer.

## INTRODUCTION

Breast cancer causes the most frequent women cancer prevalence and mortality in the world [1]. Up to 70% breast cancer cases are driven by estrogen receptor α (ERα) and anti-estrogen based therapy bring significant survival benefits for breast cancer patients [2]. However, about half of endocrine treated patients endure relapse, making it a significant clinical problem [3]. Thus, it is urgent and necessary to understand the potential mechanisms and insight into the novel facets and modulatory factors for estrogen signaling, which could serve for the development of promising treatment strategies.

Several mechanisms were shown to account for hyper-activation of ERα and endocrine resistance in breast cancer [4–6]. Some are related to crosstalk of other oncogenic signaling including HER2, EGFR and NF-κB pathway [4, 7]. The others are associated with the modulatory factors, which could include ERα co-activators and protein modulators, including ubiquitination, SUMOlyation and phosphorylation [8–10]. However, the detailed mechanism that how ERα protein and its signaling are controlled by these modulators still remains largely unclear. As a group of ubiquitin ligases have been shown to facilitate estrogen signaling in breast cancer cell, such as BRCA1, CHIP and RNF31, it may suggest ER signaling and turnover is tightly linked to ubiquitin-proteasome system [11–14].

The ERα protein stability and turnover could be controlled by several ubiquitination manners [15]. Interestingly, the ubquitinated ERα does not necessary lead to decreased protein stability [16]. For example, ERα mono-ubiquitination causes increased protein stability and enhanced ERα signaling activity [13]. However, the cellular factors that trigger and recognize this type of modification need to be further characterized. Our current study identifies the ubiquitin associated protein SHARPIN (Shank-Interacting protein-like 1, SIPL1) as a novel ERα modulation factor. SHARPIN was firstly cloned from nerve cells and was found to endure gene amplification in several human cancers, including breast tumor [17–19]. However, its function in breast carcinogenesis and estrogen signaling remains to be addressed. Here, we identify SHARPIN to control ERα ubiquitination and stability and thereby the transcriptional regulation of ERα target genes and breast cancer cell proliferation.

## RESULTS

### SHARPIN is higher expressed in breast tumor and correlates with ERα protein in breast cancer tissues

**Figure 1:**
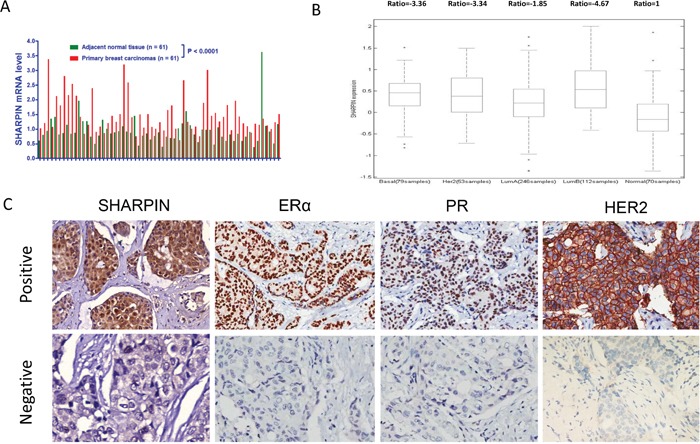
SHARPIN is higher expressed in breast tumor, correlates with ER α protein level in breast tumors **(A)** SHARPIN mRNA level comparison between paired breast tumor and adjacent normal breast tissue from TCGA database (date: 2016-09-20). **(B)** SHARPIN is higher expressed in different subtype of breast cancer samples. The SHARPIN mRNA expression data are acquired from TCGA database (date: 2016-09-20). **(C)** Examples of positive/negative SHARPIN, ERα, PR and HER2 staining in breast tumor samples were shown by X100 magnification. The statistical data of each protein marker are shown in Table 1.

By analysis of TCGA public available database (https://tcga-data.nci.nih.gov/), we observe that SHARPIN mRNA level in breast cancer tissue is higher than normal breast tissue (Figure 1A), and SHARPIN mRNA level in breast cancer tissue is more likely to be higher compared with the adjacent normal breast tissue in individual breast cancer subtype (Figure 1B). In order to analyze the correlation between SHARPIN expression and breast cancer subtype markers, 133 breast tumor tissues are collected and immunohistochemistry (IHC) is applied for examine the protein level of SHARPIN, ERα, progesterone receptor (PR), human epidermal growth factor receptor-2 (HER2). The control staining is in Figure 1C. The pathological character and lymph node status data are also collected. The IHC results show that SHARPIN expression is positively correlated with ERα in clinical samples (Table 1). Then we measure the SHARPIN expression in both ERα positive cell lines (MCF-7 and T47D) and ERα negative cell lines (BT549 and MDAMB231) by western blot. Immuno-blotting shows that SHARPIN is ubiquitously expressed in both ERα positive and negative cell lines (Supplementary Figure 1A).

**Table 1:** SHARPIN correlates with ERαprotein level in breast tumors

Clinical and molecular characteristics (Cases)		SHARPIN		
		+	-	P value
**ERα**	+	69	9	0.017
	-	39	15	
**PR**	+	45	5	0.096
	-	63	17	
**HER2**	+	42	7	0.441
	-	57	14	
**lymph node metastasis**	+	49	24	0.177
	-	59	9	
**pathological grade**	low	3	0	0.573
	medium	71	18	
	High	33	6	

### SHARPIN facilitates ERα signaling and relates to poor prognosis in ERα positive breast cancer patients

**Figure 2:**
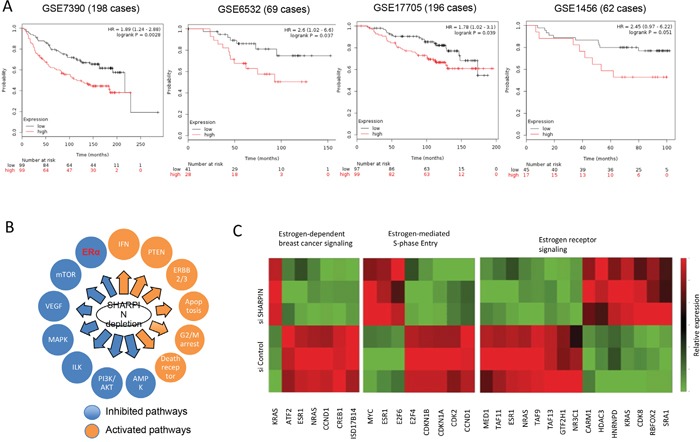
SHARPIN is related to poor prognosis in ER α positive breast cancer patients and positive correlated with ERα signaling in unbiased RNA sequencing screening **(A)** Kaplan-Meier plot showing that high SHARPIN expression correlates with relapse free survival in ER+, tamoxifen treated patients stratified for high (red) and low (black) SHARPIN expression levels in GSE7390 dataset (n = 198; optimized cut-off; Probe 220973_s_at: HR = 1.98; 95% CI: 1.24–2.88; P = 0.0028); GSE6532 dataset (n = 69; optimized cut-off; Probe 220973_s_at: HR = 2.6; 95% CI: 1.02–6.6; P = 0.037) and GSE17705 dataset (n = 196; optimized cut-off; Probe 220973_s_at: HR = 1.78; 95% CI: 1.02–3.10; P = 0.039). In GSE1456 dataset (n = 62; optimized cut-off; Probe 220973_s_at: HR = 2.45; 95% CI: 0.97–6.22; P = 0.051), although the P value shows no statistically significant, the same trend can be observed as the other two datasets (date: 2016-09-20). **(B)** Schematic graph shows significantly changed signaling by SHARPN depletion in MCF7 cells. The pathway-enrichment analysis was used by the threshold P<0.001 and fold change>2 to derive regulated genes. **(C)** The heat-map graph shows the ERα regulating genes, which is significantly changed by SHARPIN depletion in MCF-7 cells.

Through analysis of the public available breast cancer survival data ( http://kmplot.com/analysis/), we observe that SHARPIN expression correlates with poor relapse-free survival in ERα positive breast cancer patient groups of GSE 7390 dataset, GSE6532 dataset and GSE17705 dataset (Figure 2A). In GSE1456 dataset, although the P value shows no statistically significant, the same trend can be observed as the other two datasets. To approach the function of SHARPIN in breast cancer cells in an unbiased way, we analyzed changes in previously generated global gene expression profiles following SHARPIN depletion in MCF-7 breast cancer cells (Assessing number: GSE77261). The pathway enrichment analysis reveals that SHAPRIN depletion significantly changes several pathways, including ERα and PTEN (Figure 2B and Table 2). The regulatory effect of SHARPIN on PTEN/AKT was reported in previous and is observed from our RNA-sequence data [20]. By specific analysis of ERα signaling, we observe a group of ERα activating target genes are decreased, including ERα itself, while another group of ERα suppressing target genes are increased, such as CDKN1A and CDKN1B (Figure 2C and Table 3). These RNA sequence data indicate that SHARPIN might play a supportive role in ERα signaling.

**Table 2:** Related canonical pathway for activity analysis

Ingenuity Canonical Pathways	P value	Ratio	z-socre
**Interferon Signaling**	0.00	0.31	3.16
**PTEN Signaling**	0.01	0.14	1.50
**ErbB2-ErbB3 Signaling**	0.03	0.16	2.12
**Retinoic acid Mediated Apoptosis Signaling**	0.03	0.16	1.89
**Cell Cycle: G2/M DNA Damage Checkpoint Regulation**	0.03	0.16	2.12
**Death Receptor Signaling**	0.04	0.13	1.73
**UVA-Induced MAPK Signaling**	0.07	0.13	1.90
**AMPK Signaling**	0.00	0.15	-1.70
**PI3K/AKT Signaling**	0.01	0.14	-2.18
**ILK Signaling**	0.02	0.12	-2.24
**ERK/MAPK Signaling**	0.03	0.12	-1.71
**NRF2-mediated Oxidative Stress Response**	0.03	0.12	-2.31
**VEGF Signaling**	0.14	0.11	-1.67
**Estrogen-Dependent Breast Cancer Signaling**	0.16	0.11	-1.63
**mTOR Signaling**	4.92	0.10	-1.81

**Table 3:** estrogen signaling related genes changed by SHARPIN knockdown

		Fold Change	Expected change by estrogen
Estrogen-dependent breast cancer signaling	ATF2	-1.785	Up
Estrogen-dependent breast cancer signaling	CCND1	-1.808	Up
Estrogen-dependent breast cancer signaling	CREB1	-1.815	Up
Estrogen-dependent breast cancer signaling	ESR1	-1.766	up
Estrogen-dependent breast cancer signaling	HSD17B14	-1.814	down
Estrogen-dependent breast cancer signaling	KRAS	1.814	up
Estrogen-dependent breast cancer signaling	NRAS	-1.801	up
Estrogen receptor signaling	CARM1	1.795	none
Estrogen receptor signaling	CDK8	1.801	none
Estrogen receptor signaling	ESR1	-1.766	Up
Estrogen receptor signaling	GTF2H1	-1.812	up
Estrogen receptor signaling	HDAC3	1.782	up
Estrogen receptor signaling	HNRNPD	1.784	up
Estrogen receptor signaling	KRAS	1.814	up
Estrogen receptor signaling	MED1	-1.798	none
Estrogen receptor signaling	NR3C1	-1.759	up
Estrogen receptor signaling	NRAS	-1.801	up
Estrogen receptor signaling	RBFOX2	1.815	down
Estrogen receptor signaling	SRA1	1.796	none
Estrogen receptor signaling	TAF9	-1.815	up
Estrogen receptor signaling	TAF11	-1.795	none
Estrogen receptor signaling	TAF13	-1.798	none
Estrogen-mediated S-phase Entry	CCND1	-1.808	up
Estrogen-mediated S-phase Entry	CDK2	-1.811	up
Estrogen-mediated S-phase Entry	CDKN1A	1.8	Down
Estrogen-mediated S-phase Entry	CDKN1B	-1.79	Down
Estrogen-mediated S-phase Entry	E2F4	1.806	up
Estrogen-mediated S-phase Entry	E2F6	1.769	up
Estrogen-mediated S-phase Entry	ESR1	-1.766	Up
Estrogen-mediated S-phase Entry	MYC	-1.816	Up

### SHARPIN controls ERα signaling in breast cancer cells

**Figure 3:**
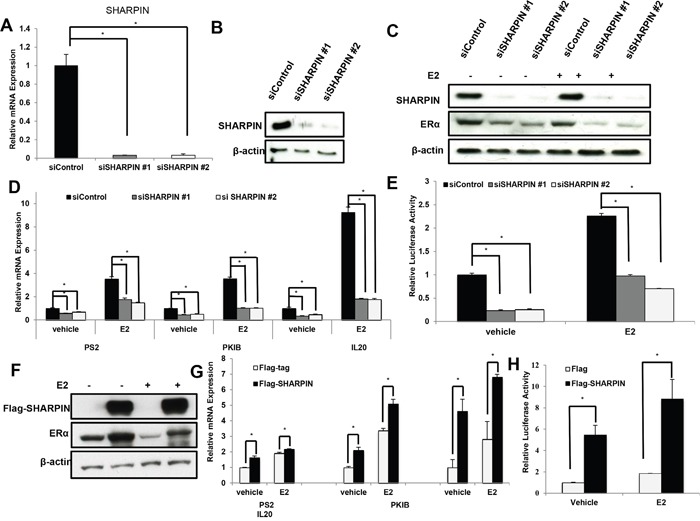
SHAPRIN controls ERα signaling activity in breast cancer cells **(A)** and **(B)** SHARPIN depletion effect by two different siRNA oligos. MCF-7 cells are transfected with siSHARPIN or siControl. After 48 h, SHARPIN mRNA and protein levels are determined by Western blot analysis. β-actin was used as internal control. **(C)** SHARPIN depletion effect on ERα protein level by two different siRNA oligos. MCF-7 cells were transfected with siSHARPIN or siControl. After 48 h, cells were treated with either ethanol or 10 nM estradiol for 6 h. SHARPIN and ERα protein levels were determined by Western blot analysis. β-actin was used as internal control. **(D)** SHARPIN depletion decreases ERα target genes using two different siRNA oligos. MCF-7 cells were transfected with siSHARPIN or siControl. After 48 h, cells were treated with either ethanol or 10 nM estradiol for 6 h. Total RNA was prepared and the expression of the endogenous ERα target genes, PS2, PKIB, and IL20 were determined by real-time PCR. Shown are the results from three experiments. *P < 0.05 for siSHARPIN versus siControl. **(E)** SHARPIN depletion affects ERE-luciferase activity in MCF7 cells. MCF7 cells were transfected with siSHAPRIN or siControl together with ERE luciferase reporter plasmid. Cells were treated with 10 nM estradiol or vehicle. Luciferase activity was measured 48 h after transfection. Shown are the results from three experiments. *P < 0.05 for siSHARPIN versus siControl. **(F)** SHARPIN overexpression effect on ERα protein level. MCF-7 cells were transfected with SHARPIN plasmid or Flag empty vector. After 48 h, cells were treated with either ethanol or 10 nM estradiol for 6 h. SHARPIN and ERα protein levels were determined by Western blot analysis. β-actin was used as internal control. **(G)** SHARPIN overexpression increases ERα target genes. MCF-7 cells were transfected with SHARPIN plasmid or Flag empty vector. After 48 h, cells were treated with either ethanol or 10 nM estradiol for 6 h. Total RNA was prepared and the expression of the endogenous ERα target genes, PS2, PKIB, and IL20 were determined by real-time PCR. Shown are the results from three experiments. *P < 0.05 for SHARPIN overexpression versus Control. **(H)** SHARPIN depletion affects ERE-luciferase activity in MCF7 cells. MCF-7 cells were transfected with SHARPIN plasmid or Flag empty vector, together with ERE luciferase reporter plasmid. Cells were treated with 10 nM estradiol or vehicle. Luciferase activity was measured 48 h after transfection. Shown are the results from three experiments. *P < 0.05 for SHARPIN overexpression versus Control.

In order to confirm the SHARPIN function in ERα signaling, we deplete SHARPIN by two individual siRNAs (Figure 3A and 3B). SHARPIN depletion significantly decreases ERα protein level in MCF7 and T47D cells (Figure 3C and Supplementary Figure 1B). However, SHARPIN depletion does not significantly change PR and HER2 protein level (Supplementary Figure 1C and 1D). In order to rule out the P53 regulatory effect on ERα protein, we double deplete both SHARPIN and P53. SHARPIN and P53 double depletion still manifests ERα protein decrease, which indicates that the regulatory role of SHARPIN on ERα protein is independent of P53 pathway (Supplementary Figure 1E). By examining ERα target genes, we find that SHARPIN depletion significantly decreases ERα classic target genes (PS2, PKIB and IL20) in both vehicle and estradiol (E2) treated condition (Figure 3D). By checking the estrogen response element (ERE) activity, it shows that inhibition of SHARPIN decreases ERE luciferase activity under both vehicle and E2-treated condition (Figure 3E). Consistent with this, SHARPIN overexpression significantly increases ERα protein level, ERα target genes and ERE luciferase activity (Figure 3F–3H). However, we do not observe the regulation of SHARPIN expression in both E2 and tamoxifen treatments (Supplementary Figure 2A and 2B).

### SHAPRIN promotes E2-stimulated proliferation in breast cancer cells

**Figure 4:**
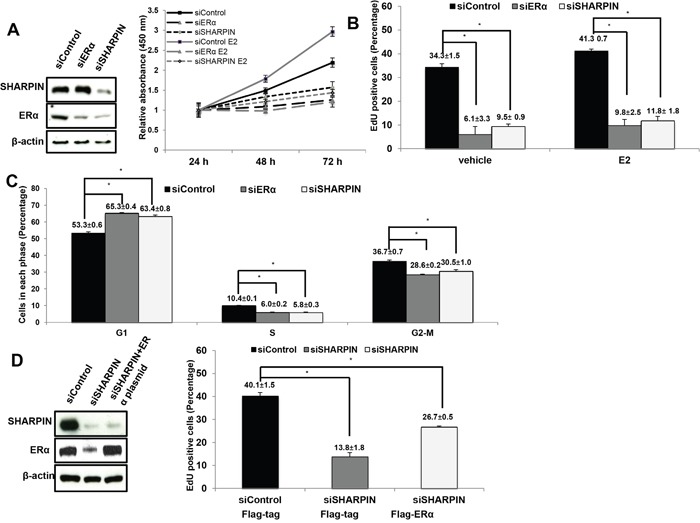
SHAPRIN facilitates estrogen-stimulated cell proliferation in breast cancer cells **(A)** The WST-1 assay was used to determine the cellular metabolic activity at indicated time points after transfection. MCF7 cells were transfected with siSHARPIN and siControl. After 24 h, cells were seeded into the 96 well plates with or without estradiol treatment. ERα depletion was used as the positive control. These experiments were done in triplicates. All values are mean ± s.d. (n = 3, *P < 0.05). SHARPIN and ERα protein levels were determined by Western blot analysis. β-actin was used as internal control. **(B)** SHAPRIN knockdown decreases cell proliferation in MCF-7 cells as determined by EdU incorporation. MCF7 cells were transfected with siSHARPIN and siControl. ERα depletion was used as the positive control. Cells were treated with or without estradiol. EdU was added at a concentration of 10 μM and incubated for 1 h. The cells were subject to FACS analysis. All values are mean ± s.d. (n = 3, *P < 0.05). **(C)** SHARPIN knockdown induces G1 cell cycle arrest and inhibits estradiol-stimulated cell proliferation. The effects of SHARPIN knockdown were compared with siControl. ERα knockdown was used as the positive control. Cells were treated with estradiol or vehicle for 24 h. The proportion of cells in each phase was measured by fluorescent-activated cell sorting. All values are mean ± s.d. (n = 3, *P < 0.05). **(D)** The decreased cell proliferation by SHARPIN knockdown could be partially rescued by ERα over-expression. MCF7 cells were transfected with siSHARPIN and siControl. After 24 h, siSHARPIN group was transfected with ERα plasmid, while the other groups were transfected with empty vector. EdU was added at a concentration of 10 μM and incubated for 1 h. The cells were subject to FACS analysis. All values are mean ± s.d. (n = 3, *P < 0.05).

To investigate the role of SHARPIN in cell proliferation, we utilize ERα-positive breast cancer cell MCF7 as a model. We deplete SHARPIN in MCF7 cells, while ERα depletion is used as the positive control (Figure 4A). The WST-1 assay shows that depletion of SHARPIN significantly decreases cell proliferation compared with siControl group (Figure 4A). Besides, SHARPIN depletion also decreases cell growth in T47D cells (Supplementary Figure 2C). The ethynly-deoxyuridine (EdU) incorporation assay shows that depletion SHARPIN decreases EdU positive cells, which is similar as ERα depletion (Figure 4B, Supplementary Figure 3). In order to confirm the phenotype, flow cytometry based propidium iodide (PI) staining is used for cell cycle analysis. Both SHARPIN depletion and ERα depletion increase the cells in G1 phase (Figure 4C, Supplementary Figure 4A). We further perform the rescue experiment by transfection ERα into the SHARPIN depletion cells. ERα overexpression could at least partially rescue the decrease of EdU positive cell by SHARPIN knocking down (Figure 4D, Supplementary Figure 4B).

### SHAPRIN associates with ERα both in cytoplasm and nuclear, but does not transcriptionally facilitates ERα signaling

**Figure 5:**
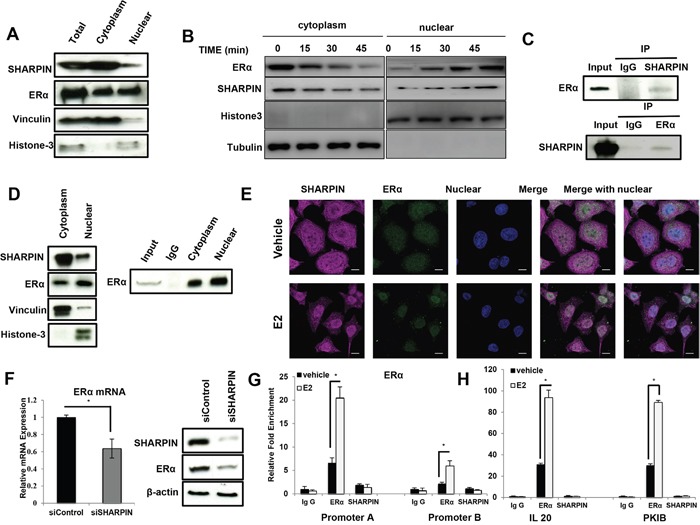
SHARPIN associates with ERα both in the cytoplasm and nuclear, but does not transcriptionally regulate ERα and its target genes **(A)** SHAPRIN protein is mainly localized in the cytoplasm. The subcellular protein fractionation kit (Thermo scientific, 78840) was used for cytoplasm and nuclear separation. Vinculin and Histone-3 were used for cytoplasm and nuclear control. **(B)** SHARPIN protein could shuttle into nuclear by estradiol treatment. Cells were subject to estradiol treatment for indicated time points (15min, 30min and 45min). The subcellular protein fractionation kit (Thermo scientific, 78840) was used for cytoplasm and nuclear separation. Vinculin and Histone-3 were used for cytoplasm and nuclear control. **(C)** Co-IP assay reveals association between endogenous SHAPRIN and ERα in MCF7 cells. **(D)** SHARPIN protein could associate with ERα both in the cytoplasm and nuclear. The subcellular protein fractionation kit (Thermo scientific, 78840) was used for cytoplasm and nuclear separation. Vinculin and Histone-3 were used for cytoplasm and nuclear control. **(E)** Intracellular localization analysis of SHARPIN and ERα by immunofluorescence assay. MCF7 cells were treated with 10 nM estradiol or vehicle for 30 min before fixation. Intracellular localization of SHAPRIN (pink) and ERα (green) were shown. Nuclei (blue) were stained with 4’, 6-diamidino-2-phenylindole (DAPI). **(F)** SHARPIN depletion effect on ERα mRNA and protein level. The total mRNA and protein were collected 24 h after siSHARPIN transfection. **(G)** ChIP assay for ERα and SHARPIN recruitment to ERα promoter A and B. MCF7 cells were treated with 10 nM estradiol or vehicle for 30 min before fixation. Rabbit Ig G was used as the negative control, while ERα antibody was used as the positive control. The primer sequences were shown in Supplementary Table 1. All values are mean ± s.d. (n = 3, *P < 0.05). **(H)** ChIP assay for ERα and SHARPIN recruitment to IL20 and PKIB promoter regions. MCF7 cells were treated with 10 nM estradiol or vehicle for 30 min before fixation. Rabbit IgG was used as the negative control, while ERα antibody was used as the positive control. The primer sequences were shown in Supplementary Table 1. All values are mean ± s.d. (n = 3, *P < 0.05).

The cytoplasmic and nuclear separation assay shows that SHARPIN is mainly localized in the cytoplasm (Figure 5A). Interestingly, 20 minutes of E2 treatment could promote the trans-location of SHARPIN from cytoplasm into nucleus (Figure 5B). Immuno-precipitation (IP) shows that SHARPIN could associate with ERα both in the cytosol and nucleus (Figure 5C and 5D). The trans-location of SHARPIN is further confirmed by immunocytochemistry (Figure 5E). However, there are two possibilities for SHARPIN effect on ERα signaling-transcriptional regulation or protein regulation. We deplete SHARPIN and collect mRNA and protein as early as 24 h. The ERα mRNA and protein level are both decreased at this time point (Figure 5F). Then, this give rises to two possible models that SHARPIN may regulate ERα gene expression by co-occupied with ERα on the target gene promoter regions. Seven promoters are identified from ERα gene, while it is already known that promoter A and B is commonly active in MCF-7 cells [21]. We perform chromatin immuno-precipitation (ChIP) by SHARPIN antibody, while ERα based ChIP is used as the positive control. However, SHARPIN based ChIP fails to detect the promoter binding of ERα and its classical target genes, such as IL20 and PKIB (Figure 5G and 5H).

### SHARPIN stabilizes ERα protein possibly through mono-ubiquitinating ERα at K302/303 sites

**Figure 6:**
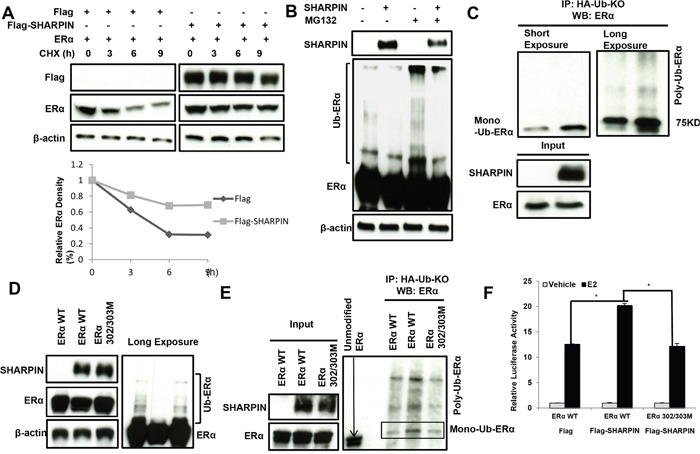
SHAPRIN stabilizes ERα by promoting its mono-ubiquitination at K302/303 sites **(A)** SHARPIN increases ERα half-life in HEK293 cells. HEK293 cells were transfected with 0.5 μg ERα plasmid and 0.5 μg Flag-tag or Flag-SHARPIN plasmids. After 24 h, cells were treated with 100 μM cycloheximide/vehicle for indicated times. Cell lysates were prepared for Western blot analysis. The results are representative for three independent experiments. **(B)** SHARPIN prohibits ERα poly-ubiquitination. HEK293 cells were transfected with 0.5 μg ERα plasmid and 0.5 μg Flag-tag or Flag-SHARPIN plasmids. After 24 h, cells were treated with 10uM MG132 or vehicle for 6 hours. Cells were directly harvested and Western blot analysis using ERα antibody was used to detect ubiquitinated ERα forms. The predicted molecular weight of polyubiquitinated ERα is indicated. **(C)** Direct evidence for ERα mono-ubiquitination by SHARPIN protein. HEK293 cells were transfected with 0.5 μg ERα plasmid, 0.5 μg HA-Ub-KO plasmid and 0.5 μg Flag-tag or Flag-SHARPIN plasmids. The cell extracts were immuno-precipitated with HA antibody. Mono-ubiquitinated ERα was detected via western blotting analysis. **(D)** The poly-ubiquitination inhibition effect by SHARPIN depends on ERα 302/303 sites. HEK293 cells were transfected with 0.5 μg ERα plasmid (or ERα 302/303AA mutant) and 0.5 μg Flag-tag or Flag-SHARPIN plasmids. After 24 h, cells were treated with 10 uM MG132 or vehicle for 6 hours. Cells were directly harvested and Western blot analysis using ERα antibody was used to detect ubiquitinated ERα forms. The predicted molecular weight of polyubiquitinated ERα is indicated. **(E)** Direct evidence for ERα mono-ubiquitination at 302/303 sites. HEK293 cells were transfected with 0.5μg ERα plasmid (or ERα 302/303AA mutant), 0.5μg HA-Ub-KO plasmid and 0.5μg Flag-tag or Flag-SHARPIN plasmids. The cell extracts were immuno-precipitated with HA antibody. Mono-ubiquitinated ERα was detected via western blotting analysis. **(F)** ERα-lysine 302/303 is necessary for the SHARPIN mediated ERα signaling. Flag-SHARPIN or Flag control and ERα wild type or ERα 302/302AA were transfected in the indicated combinations in HEK293 cells. 24 h before measurement, cells were transfected with an ERE luciferase reporter. After 18 h, cells were treated with 10 nM estradiol or vehicle, and an ERE-luciferase assay was carried out 6 h after E2 addition. Shown values represent mean ± s.d. (n = 3), which is representative for three independent experiments. * - *P* < 0.05 for Flag-SHARPIN group versus control, according to t-test.

Since SHARPIN does not regulate ERα in transcription level, we infer that SHARPIN might regulate ERα through post-translational mechanisms. Upon inhibition of protein synthesis by cycloheximide, the presence of SHARPIN significantly prolongs the half-life of ERα protein in HEK293 cells (Figure 6A, Supplementary Figure 5). With the treatment of proteasome inhibitor MG132, we observe the decrease of expected poly-ubiquitin chains in the presence of SHARPIN (Figure 6B). By applying ubiquitin plasmid with all lysine mutants (Ub KO), we find that SHARPIN increases the mono-ubiquitinated ERα (Figure 6C). This suggests that SHARPIN might stabilize ERα by mono-ubiquitination. Since the amino acid lysine 302 and 303 sites are the frequently reported sites involving in mono-ubiquitination, we mutate the two sites into alanine. Figure 6D shows that SHARPIN could decrease the poly-ubiquitin chain in wild type ERα which could be rescued in ERα with K302/303 mutation. The Ub KO based immuno-precipitation shows that SHARPIN increases wild type ERα mono-ubiquitination, while it has decreased mono-ubiquitination chain in ERα 302/303 mutant (Figure 6E). In consistent with this, even SHARPIN increases ERE luciferase activity in wild type ERα, the ERα 302/303 mutations has impaired response to SHARPIN (Figure 6F).

## DISCUSSION

In our study, we demonstrate that one ubiquitin binding protein SHARPIN, which is correlated with ERα expression in clinical breast tumor samples, potentiates ERα signaling activity and promotes tamoxifen resistance through post-translational modifications. SHARPIN stabilizes ERα protein and prohibits its poly-ubiquitination, possibly by inducing ERα mono-ubiquitination at 302/303 sites.

The relationship between estrogen signaling and breast cancer was revealed since 1934 [22]. The further discovery showed the significant benefit for ERα antagonists in breast cancer treatments [23]. However, endocrine resistance is still a big problem for ERα target therapy [24]. Interestingly, most of the endocrine resistance tumors are still ERα positive. Breast cancer cells get endocrine resistance through ERα modifications, including phosphorylation, ubiquitination and acetylation, to overcome the blocking effect by ERα antagonists [25, 26]. Even the post-translational modifications spread in ERα protein, only a few sites affect the ERα function, such as the modifications in the hinge domain of ERα [27–29]. The modifications in the hinge domain of ERα could affect the protein conformation and subsequent ligand/DNA binding affinity [30]. Quite a few studies have shown several key modification sites for ERα activity, including lysine 302/303 and serine 305 [31, 32]. For example, the p21-activated kinase family proteins were shown to facilitate ERα signaling by phosphorylating S305 site, increasing protein stability and promoting tamoxifen resistance [8, 33]. Besides, mono-ubiqutiination on K302/303 sites was also shown to promote ERα protein stability and ERα signaling activity [34]. Here, our research firstly identified the ubiquitin binding protein SHARPIN and its possible link for K302/K303 modification with tamoxifen resistance. We believe that the novelly found ERα modifiers will not only help to understand the complexity of ERα signaling, but also increase the knowledge of less known ubiquitin binding proteins in nuclear receptor function.

SHARPIN protein was firstly found from nerve cells and characterized as the SHARK-interaction protein [17]. Further studies revealed that SHARPIN could associate with integrin and inhibit cell migration [35]. One of the most important finding is that SHARPIN is necessary for intact immune response. SHARPIN was shown to be the component of linear ubiquitin assembly complex (LUBAC) and facilitate NF-κB signaling transduction [36]. SHARPIN depletion will impair the linear ubiquitination of IKKr, which will cripple the P65/P50 translocation into the nuclear [37]. Phenotypically, SHARPIN knockout mice present with chronic proliferative dermatitis and impaired B and T cell development [38–40]. However, less is known about SHARPIN function in human cancer, even it endure a high gene amplification in TCGA database, such as pancreatic cancer and breast cancer ( http://www.cbioportal.org). Interestingly, our study reveals that SHAPRIN is not only higher expressed in breast cancer, but also correlates with ERα protein level and poor tamoxifen response. Besides, our study further shows SHARPIN involves in ERα mono-ubiquitination at K302/303 sites, which is a novel ubiquitination manner for SHARPIN protein. Although our previous study showed another component-RNF31 could also promote ERα mono-ubiquitination in breast cancer cells, the RNF31 modification effect on ERα is not dependent on the Ubiquitin-associated domain (UBA), which is necessary for LUBAC formation. Thus, we believe that SHARPIN mono-ubiquitinates ERα, which is independent of RNF31 or LUBAC function [13].

In the study, we examine the role of SHARPIN in ERα positive breast cancer cells. SHARPIN is shown to associate ERα protein and prolong its stability via mono-ubiquitination at ERα hinge domain. Since the ERα signaling is required for breast cancer proliferation, modulation of ERα protein could be an approach to inhibit breast cancer cell progression and restore endocrine resistance. Besides, our unpublished data also shows SHARPIN promotes several oncogenic pathways, including hypoxia induced factors and AKT pathways. In all, SHARPIN could be a promising therapeutic strategy for breast cancer treatment.

## CONCLUSIONS

This study identifies the first time, the ubiquitin binding protein SHARPIN as a modulator of ERα signaling in human breast cancer cells. Importantly, SHARPIN depletion could rescue tamoxifen sensitivity, hamper estrogen-dependent cell proliferation and decrease ERα signaling in multiple breast cancer cell lines. As a novelly discovered modulator for ERα signaling, SHARPIN could be a promising target to overcome endocrine therapy resistance.

## MATERIALS AND METHODS

### Cell culture

MCF-7 and HEK293 cells were cultured in DMEM (Invitrogen, Carlsbad, CA) supplemented with 10% fetal bovine serum (FBS) and 1% penicillin/streptomycin (Invitrogen) at 37°C in a humidified atmosphere of 5% CO_2_ in air. T47D cells were cultured in RPMI 1640 (Invitrogen) supplemented with 10% FBS (GIBCO) and 1% penicillin/streptomycin.

### Plasmids

SHARPIN (pcDNA-Flag-SHARPIN) construct was kindly presented from Dr. Kazuhiro Iwai and was previously described. The pcDNA3-ERα plasmid, HA-ubiquitin-KO plasmid, the ERE-TK-luciferase reporter and the pRL-TK control were described in previous study [13]. The ERα 302/303 mutants (lysine to alanine) were described in previous paper [16, 37].

### siRNA and plasmids transfection

Cells were transfected with 50 nM siRNA. SHARPIN siRNAs sequences were shown here: SHARPIN siRNA #1: CUGCUUUCCUCUACUUGCUdTdT; siRNA #2: GCUUUCCUCUACUUGCUGUdTdT. p53 siRNA and control siRNA are Stealth Select siRNA (Invitrogen). INTERFERin transfection reagent (Polyplus Transfection, 409-10) was used according to the manufacturer's protocol. Plasmids were transfected by Lipofectamin 2000 (1662298, Invitrogen).

### RNA extraction and real-time PCR analysis

RNeasy kits were used to extract total RNA (Qiagen). Real-time PCR was performed as previously described. 36B4 was used as internal control. Primer sequences for Real-time PCR are provided in Supplementary Table.

### Quantification of cell viability

MCF-7 and T47D cells were transfected with siSHARPIN or siControl in 24-well plates. After 24 h, the cells were seeded into 96-well plates. Estrogen and vehicle were added in each group. Cell numbers were determined using the WST-1 cell proliferation reagent as previously described [8].

### Flow cytometry

For ethynly-deoxyuridine (EdU) labeled DNA stain, cells were transfected with siSHARPIN, siERα and siControl. After 24 h, 10 nM estradiol or vehicle was added for another 24 h. Then 10 μM EdU was added into each plate for the last 60 min. For propidium iodide staining, MCF7 cells were seeded into 10-cm dishes. After 24 h, cells were transfected with siSHARPIN, siERα and siControl. After another 24 h, cells were fixed with 70% ethanol for 30 min and stained with propidium iodide. For the ERα rescue experiment, MCF7 cells were seeded into 10-cm dishes. After 24 h, cells were transfected with siSHARPIN, and siControl. After another 24 h, siSHARPIN group was transfected with 5 ug ERα plasmid, while other groups were transfected with 5 ug Flag vector. The BD LSR II flow cytometer (BD Bioscience) was used to measure the flow fluorescence intensity.

### Western blotting

Cells were lysed with RIPA lysis buffer. Anti-ERα (HC-20, SC543) was from Santa Cruz Biotechnology. Anti-SHARPIN (AB69507), and anti-FLAG (M2, ab48763) were acquired from Abcam. Anti-actin (8H10D10) was acquired from Cell Signaling Technology.

### Immunoprecipitation

Immunoprecipitation was performed as previous described [41]. 100 ug cell lysls were pre-cleared with Rabbit IgG for 2 h and subsequently incubated with SHARPIN antibody (AB69507) over night, while rabbit Ig G was used as the negative control. The bounded protein was analyzed by ERα antibody (1DO5, santa cruz). For the overexpression experiment, HEK293 cells were transfected with 5 ug Flag-SHARPIN and ERα plasmid in 10 cm dish. Cell lysates were pre-cleared with IgG and subsequently incubate with Flag antibody or ERα antibody, while rabbit IgG was used as the negative control. The bound proteins were analyzed by western blotting.

### Protein stability assays

HEK293 cells (10^5^) were seeded into 24 well plates and transfected with 0.5 ug ERα plasmid together with 0.5 ug Flag-SHARPIN or empty Flag vector. After 48 h, cells were treated with 100 uM cycloheximide for indicated time points. Samples were subject to western blot for ERα degradation.

### Analysis of protein ubiquitination

HEK293 cells were transfected with 4 ug ERα (or ERα 302/302AA mutant) plasmid together with 4 ug Flag-SHARPIN or empty Flag vector. After 48 h, cells were treated with 10 uM MG132 or ethanol for 6 h. Cells were directly harvested. The poly-ubiquitination of ERα was detected by western blotting analysis.

### Mono-ubiquitination detection assay

To directly detect the enriched mono-ubiquitinated ERα from the cell extracts, HEK293 cells were transfected with 4 ug HA-UB-KO plasmid, 4 ug ERα (or ERα 302/302AA mutant) together with 4 ug Flag-SHARPIN or Flag-vector. After 48 h, total protein was extracted and pre-cleared by 20ul protein A (santa cruz, SC-2001) for 2 h. The supernatant was corrected and immuno-precipitated by HA antibody. Western blot with rabbit anti-ERα antibody was performed to detect mono-ubiquitinated ERα.

### Luciferase assay

The luciferase activity was done using the Dual-Luciferase Reporter kit (Promega, Germany). The ERE luciferase reporter was transfect together with renilla plasmid into the cells. The luciferase activity was measured after 24 h.

### Chromatin immuno-precipitation (ChIP) assay

ChIP assay was performed as our previous described [8]. MCF7 cells were treated with vehicle or 10 nM estradiol for 30 minutes before crosslinking. The antibodies were used as follows: SHARPIN (AB69507, abcam), ERα (HC20, santa cruz) and rabbit IgG (sc2027, santa cruz). The sequences for ChIP primers were shown in Supplementary Table.

### Immunofluorescence assay

The immunofluorescence assay was described in detail in our previous study. MCF-7 cells were treated with estradiol or vehicle for 30 min before being fixed with 4% paraformaldehyde in PBS for 10 min, permeabilized with 0.2% Triton X-100 for 5 min, and blocked by 5% BSA in PBS for 1 h. A rabbit anti-SHARPIN polyclonal antibody and mouse anti-monoclonal antibodies were used, followed by Alexa Flour 647 (Invitrogen) anti-rabbit antibody and FITC-conjugated anti-mouse antibodies (Jackson Immuno-Research, West Grove, PA).

### RNA sequence analysis

The global gene expression analysis was based on RNA sequencing platform from BGI (Beijing Genomic Institute). The RNA sequence data are deposited in the Gene Expression Omnibus (GEO) database (Assessing number: GSE77261). Analysis was performed for differentially expressed genes (P < 0.01 and fold change > 2) by Ingenuity Pathway Analysis (IPA).

### Analysis of gene expression in publicly available data sets

Analysis of SHARPIN expression in 62 paired normal breast tissues and breast cancer samples from The Cancer Genome Atlas (TCGA) was carried out in the statistical environment R. The relapse-free survival data of tamoxifen-only treated patients were acquired from KMPLOT database ( http://kmplot.com/analysis/).

### Clinical breast tumor samples

One hundred and twenty two formalin-fixed paraffin-embedded breast cancer samples were collected from the Department of Pathology, Shandong Qilu Hospital. All the breast tumors samples were examined by ERα status, PR status, HER2 status by pathological specialists. The pathological grade plus lymph node metastasis status of each sample was also examined by pathological specialists.

### Statistics

Student's t-test, Pearson correlation coefficient and Cox regression analysis were used for comparisons. A P-value of < 0.05 was considered to be significant.

## CONSENT

### Ethics, consent and permissions

This study was reviewed and approved by the Ethical Board at the Qilu Hospital of Shandong University with written informed consent from all the patients.

### Consent for publication

Not applicable.

### Availability of supporting data

Additional data are available as Supplementary Information.

## SUPPLEMENTARY MATERIALS FIGURES AND TABLES




