# Supplementary material for: SHARPIN stabilizes estrogen receptor α and promotes breast cancer cell proliferation
Source: Oncotarget. 2017 Aug 19;8(44):77137–51. doi: 10.18632/oncotarget.20368 (PMC5652769; doi:10.18632/oncotarget.20368)
Supplement: Supplementary file 1 [file oncotarget-08-77137-s001.pdf]

SHARPIN stabilizes estrogen receptor α and promotes breast cancer cell proliferation

SUPPLEMENTARY MATERIALS

Supplementary Table 1: Primer sequences for Q-PCR and ChIP assay

Gene name	Sequence
SHARPIN Forward	TAGCAGCCACCAGAGGTTAC
SHARPIN Reverse	AGCAGTCAGTAGAGGTCCCC
PKIB Forward	TCA CTG CGG GAT ATT GGC TTA
PKIB Reverse	AGC TGG TCT TCT TCC TCC TAA ACT G
IL 20 Forward	GCCAATTCCTTTClTACCATCAA
IL 20 Reverse	CCCACAATGGCATGTCATGT
ERα Forward	GCT ACG AAG TGG GAA TGA TGA AAG
ERα Reverse	TCT GGC GCT TGT GTT TCA AC
PS2 Forward	CAT CGA CGT CCC TCC AGA AGA G
PS2 Reverse	CTC TGG GAC TAA TCA CCG TGC TG
Cyclin D1 Forward	CAC GCG CAG ACC TTC GT
Cyclin D1 Reverse	GGG CGG ATT GGA AAT GAA C
ADORA1 Forward	GGA TCG ATA CCT CCG AGT CA
ADORA1 Reverse	GAG AAT CCA GCA GCC AGC TA
36B4 Forward	GGCGACCTGGAAGTCCAACT
36B4 Reverse	CCATCAGCACCACAGCCTTC
PKIB Forward CHIP	TCA CTG CGG GAT ATT GGC TTA
PKIB Reverse CHIP	AGC TGG TCT TCT TCC TCC TAA ACT G
IL20 Forward CHIP	GAT GGA TGG GAA CAC ATT GGT
IL20 Reverse CHIP	TGG TGG CGG AGC ACA AA
ERα-promoter A Forward CHIP	GGG ATC GCT CCA AAT CGA
ERα-promoter A Reverse CHIP	CTT GCC CTG ACA TTG GCT TAA
ERα-promoter B Forward CHIP	TCA GAT GCC CCC TGT CAG TT
ERα-promoter B Reverse CHIP	CAG CCA GCC ACA GAC AGC TA
18S Forward CHIP	GCTTAATTTGACTCAACACGGGA
18S Reverse CHIP	AGCTATCAATCTGTCAATCCTGTC

Supplementary Data: Breast cancer patients data

See Supplementary File 1

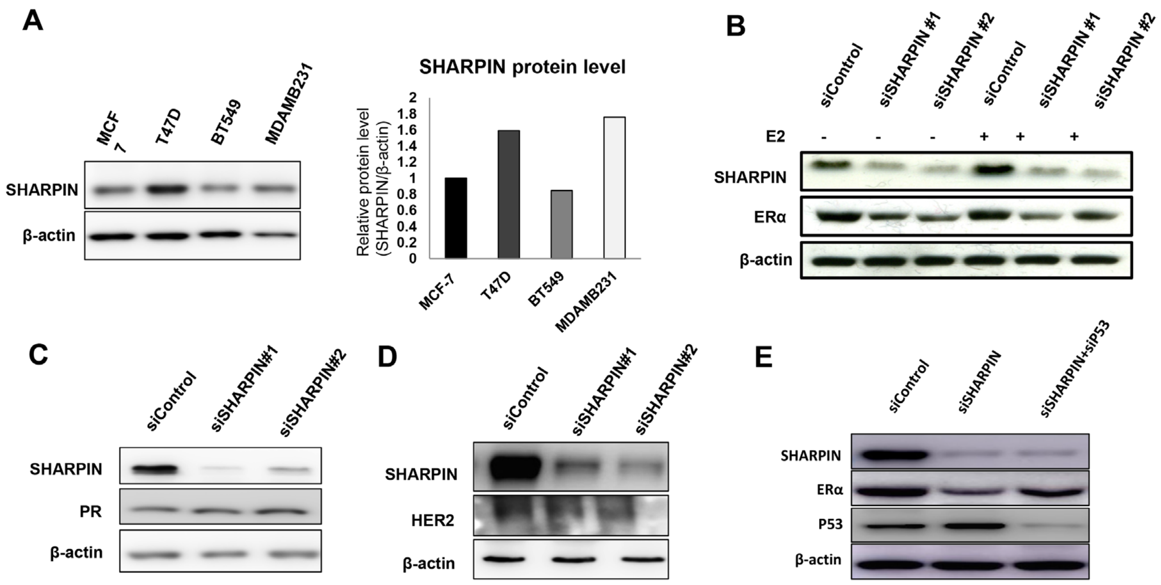

Supplementary Figure 1: (A) SHARPIN is ubiquitously expressed in both ER+ and ER- breast cancer cells. (B) SHARPIN depletion effect on ERα protein level by two different siRNA oligos in T47D cells. **(C)** SHARPIN depletion does not change PR protein level in T47D cells. **(D)** SHARPIN depletion does not change HER2 protein level in BT549 cells. **(E)** SHARPIN and P53 double depletion cannot totally rescue ERα protein level in MCF-7 cells.

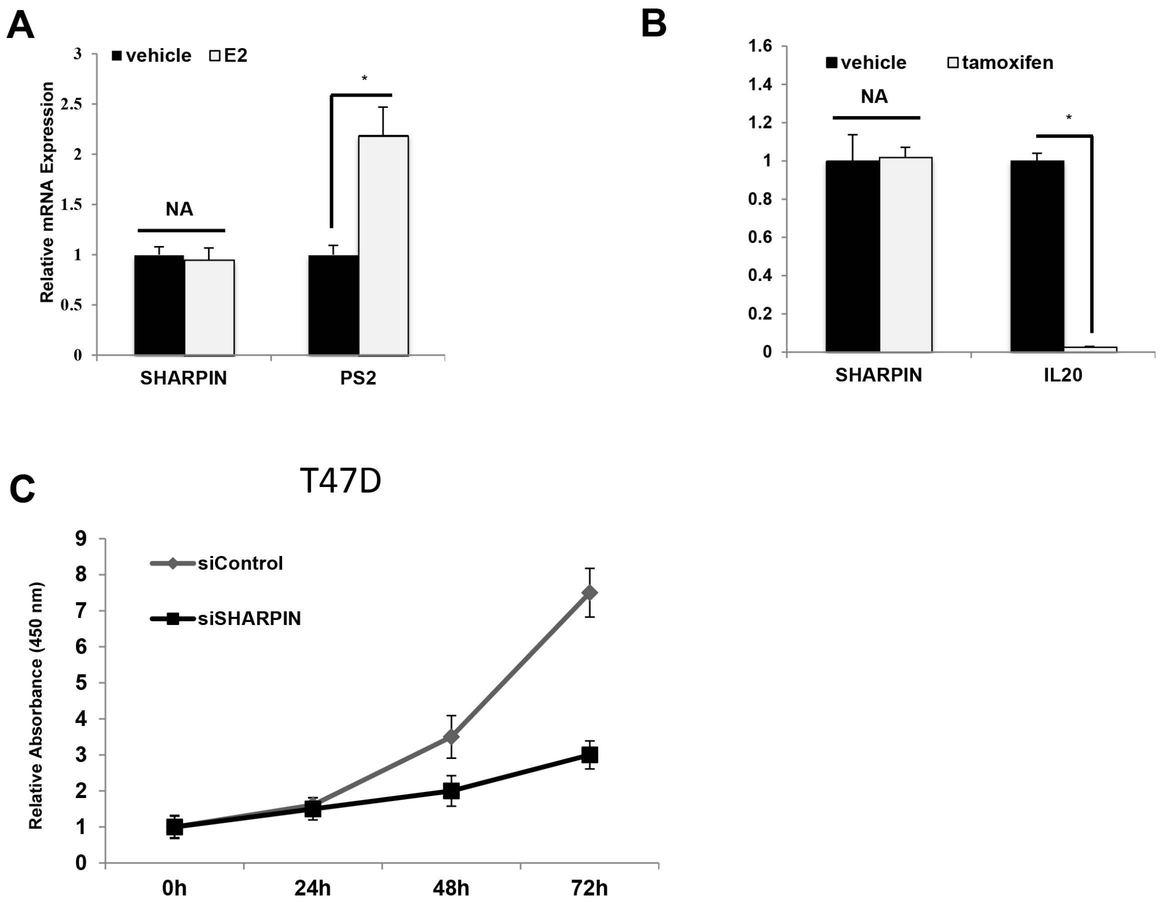

Supplementary Figure 2: (A) SHARPIN mRNA level by estradiol treatment. MCF7 cells were treated with estradiol for 6 h. PS2 was used as the positive control. **(B)** SHARPIN mRNA level by tamoxifen treatment. MCF7 cells were treated with tamoxifen for 6 h. IL20 was used as the positive control. **(C)** SHARPIN depletion decreases cell growth in T47D cells (ERα+).

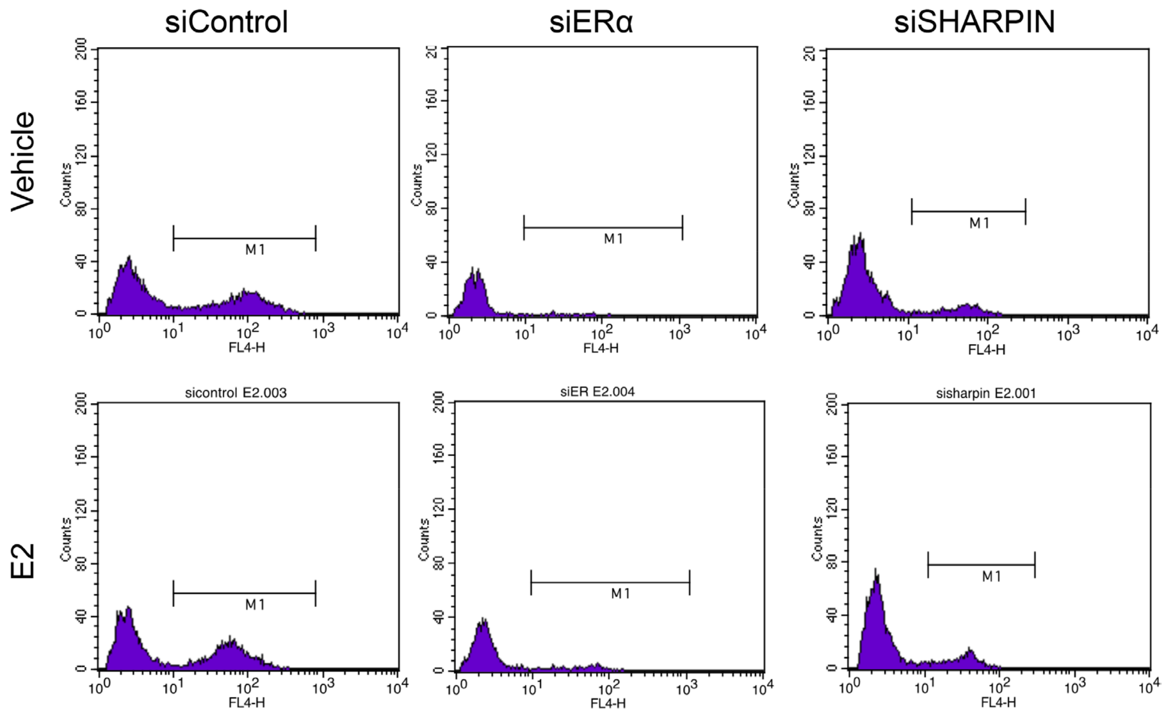

Supplementary Figure 3: The FACS histogram of Figure 4B (EdU staining by siSHARPIN, siER α and siControl).

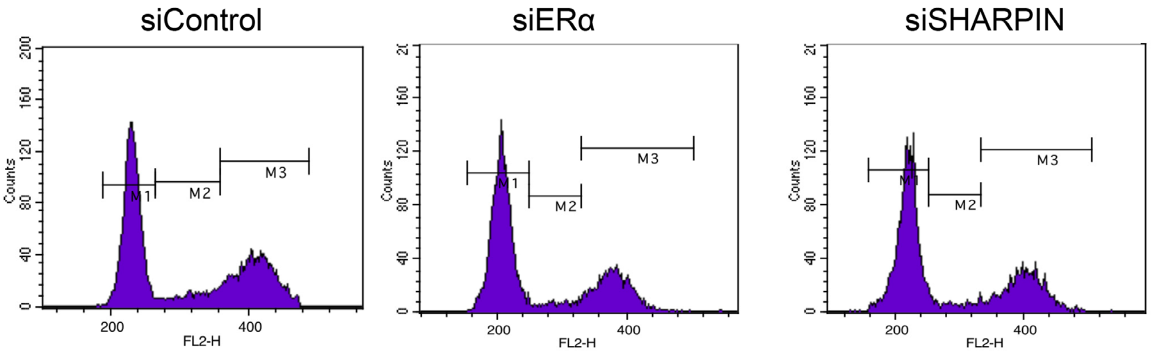

Supplementary Figure 4B

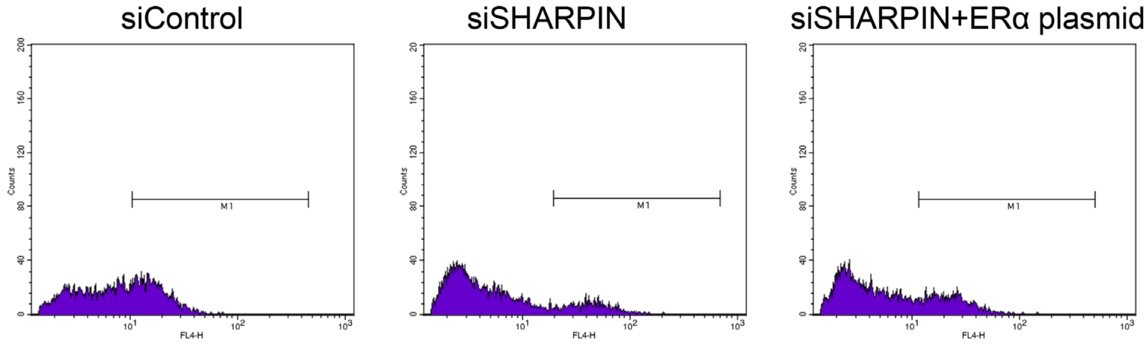

Supplementary Figure 4: **(A)** The FACS histogram of Figure 4C (PI staining by siSHARPIN, siER α and siControl). (**B)** The FACS histogram of Figure 4D (EdU staining by siSHARPIN, siSHARPIN+ER α overexpression and siControl).

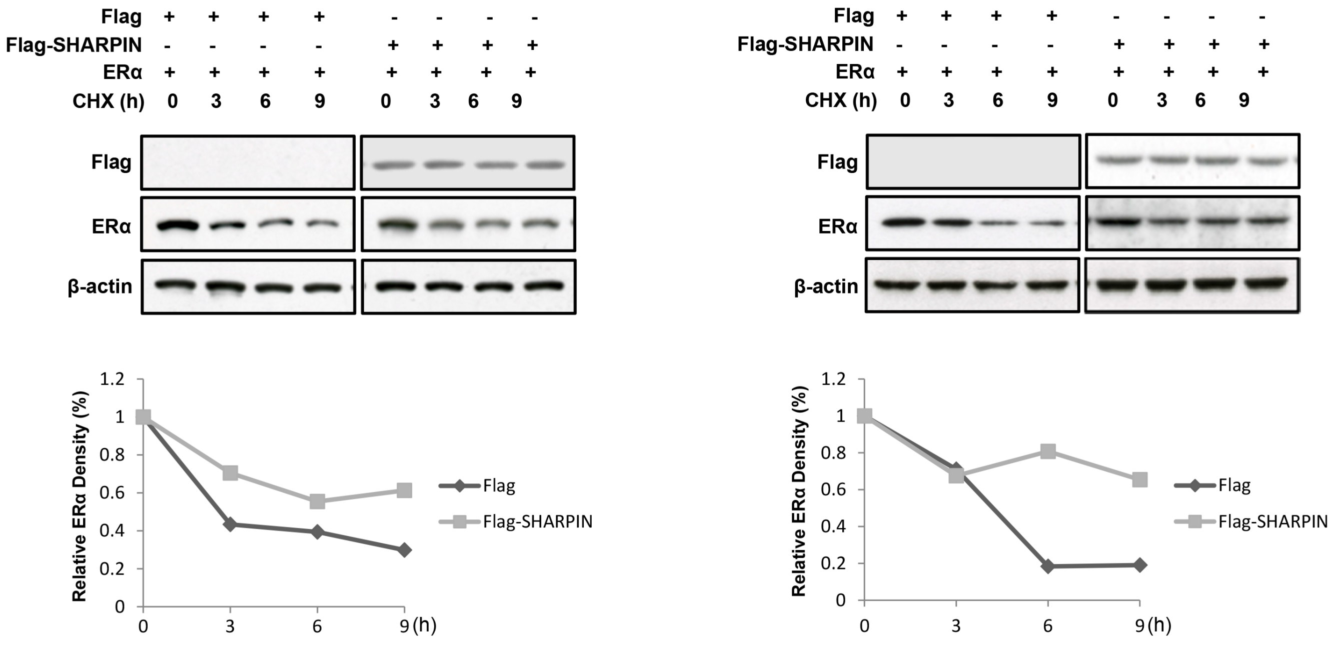

Supplementary Figure 5: Independent repeats of Figure 6A, showing that SHARPIN increases ERα half-life in HEK293 cells.
